# Pregnancy-related spontaneous coronary artery dissection: a rare cause of acute coronary syndrome in the third trimester

**DOI:** 10.1186/s12872-023-03323-7

**Published:** 2023-06-08

**Authors:** Kenny Vongbunyong, Frederick Chua, Roxana Ghashghaei

**Affiliations:** 1 Department of Medicine, University of California, 333 The City Blvd. West, Suite 400, Orange, Irvine, CA 92868 USA; 2 Department of Medicine, Division of Cardiology, University of California, Irvine, CA USA

**Keywords:** Spontaneous coronary artery dissection, Pregnancy-associated spontaneous coronary artery dissection, Coronary angiography, Percutaneous coronary intervention, Acute Coronary Syndrome

## Abstract

**Background:**

Spontaneous Coronary Artery Dissection (SCAD) is a rare cause of myocardial infarction and sudden cardiac death that is mostly seen in younger patients without significant cardiac risk factors. The mechanism by which SCAD causes an acute coronary event is related to the compromise of the coronary artery lumen as a result of hematoma within the vessel wall. In comparison to their non-pregnant counterparts, when SCAD is associated with pregnancy, it has been associated with an increased risk of life-threatening arrhythmias, cardiogenic shock, and death. The underlying mechanism behind SCAD is not yet fully understood, and despite the condition’s high mortality rate, it remains underdiagnosed.

**Case Presentation:**

Our case features a 38-year-old woman at 29 weeks of gestation presenting with chest pain that persisted despite initial management. Coronary angiography revealed a Type 2a spontaneous dissection of the left anterior descending artery. Given the risks of percutaneous coronary intervention in SCAD management and overall clinical stability, the patient was treated with conservative management.

**Conclusion:**

SCADs are a rare cause of acute coronary syndrome that can be found in patients without any prior cardiac risk factors. It is important to have a high index of suspicion when diagnosing SCADs given, they can cause life-threatening arrhythmias, cardiogenic shock, and death. This case highlights considerations that must be taken into account when treating P-SCAD, as opposed to SCAD in the postpartum period.

## Background

Spontaneous Coronary Artery Dissection (SCAD) is an often-overlooked cause of acute coronary syndrome and sudden cardiac death that mainly occurs in younger otherwise healthy patients. There is a known association between SCAD and fibromuscular dysplasia, and additionally connective tissue disorders such as Marfan’s and Ehlers-Danlos have been reported as predisposing factors for SCAD. Depending on the severity of coronary artery dissection, patients may present with symptoms ranging from chest pain to ventricular arrhythmias, or even sudden cardiac death. SCAD is usually diagnosed after angiography and is treated with medical management. Further interventions such as percutaneous coronary intervention (PCI) or coronary artery bypass grafting (CABG) are reserved for severe lesions and require evaluation on a case-by-case basis. Pregnancy-associated spontaneous coronary artery dissection (P-SCAD) occur in approximately 1.81 per every 100,000 pregnancies [1]. P-SCAD is defined as a SCAD event occurring during pregnancy or within the first 3 months of the postpartum period. When compared to non-pregnant patients with SCAD, P-SCAD patients tended to have more severe clinical manifestations and an increased risk of life-threatening arrhythmias, cardiogenic shock, and death [2]. According to the Mayo Clinic SCAD Registry, 70% of P-SCAD cases were found to occur during the first month following delivery, with 54% of cases occurring within the first week following delivery [3]. Although SCAD is perhaps the most common etiology of myocardial infarction during pregnancy, the pathophysiology behind SCAD is not yet clearly understood. Whereas the majority of P-SCAD cases occur during the postpartum period, we present a rare case of P-SCAD occurring in the third trimester of pregnancy that was medically managed.

## Case presentation

A 38-year-old nulliparous woman, G1P0 (Gravida 1 Parity 0) at 29 weeks gestation of an in vitro fertilization (IVF), presented to the emergency department with chest pain. Her medical history was pertinent for gestational hypertension, migraines, iron deficiency, and a prior abdominal myomectomy for fibroid removal. She was visiting from out of state and earlier that night she was changing her clothes when she experienced a substernal crushing chest pain that radiated to her left arm associated with diaphoresis. It did not improve despite two doses of sublingual nitroglycerin. She was hospitalized 1 week prior for similar chest pain and was treated conservatively for a clinical diagnosis of SCAD with beta blocker and aspirin. At that time echocardiogram was unremarkable and electrocardiogram (ECG) did not reveal any specific abnormalities. High sensitivity troponin peaked at 13,449 and subsequently down-trended.

**Fig. 1:**
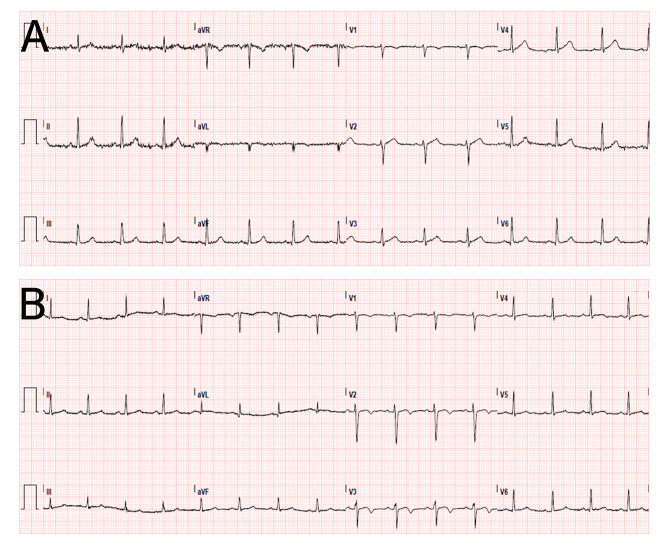
Electrocardiogram. Fig 1**a**) ECG on admission showing sinus rhythm without signs of ischemia. Fig 1**b**) ECG during recurrent chest pain, revealing sub-millimeter ST elevations in V1 and V2, and T-wave inversions in V1-V3.

On presentation in the emergency department, she was afebrile with a heart rate of 90, blood pressure 102/61, respiratory rate 20, with normal oxygen saturation on room air. Physical exam was consistent with a female at 29 weeks gestation, but was otherwise unremarkable. Initial ECG showed sinus rhythm without signs of ischemia (Fig. 1a). High sensitivity troponin was 85 ng/L (< 15 ng/L normal), repeat was 88 ng/L and subsequently it down-trended to 28 ng/L. Her chest pain persisted for another 2 h without any significant improvement despite repeated nitroglycerin and labetalol administration. The patient was admitted for telemetry monitoring in the cardiac care unit and the obstetric team followed closely for recommendations.

**Fig. 2:**
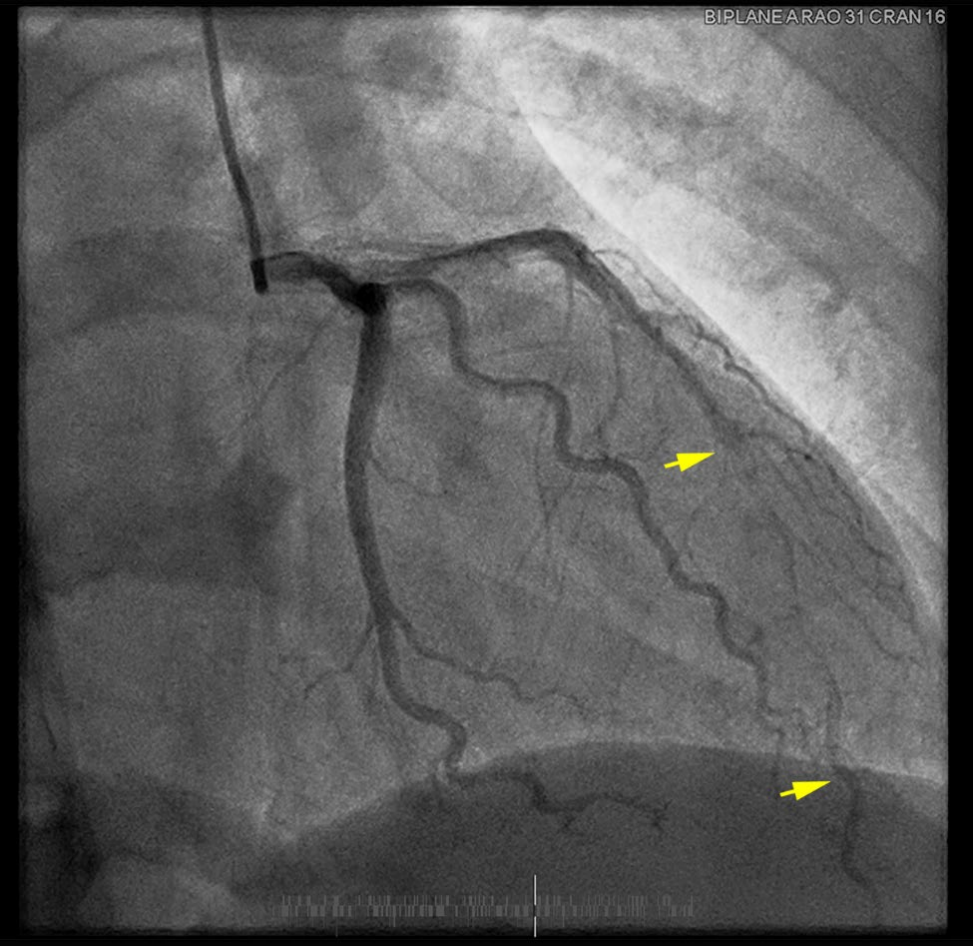
Coronary Angiography of SCAD. RAO Cranial Angiography revealing mid to distal LAD smooth diffuse stenosis consistent with Type 2a SCAD.

Transthoracic Echocardiogram showed a left ventricular ejection fraction of 59%, with impaired diastolic filling and hypokinesis of the apical lateral segment. The following day she experienced a recurrent episode of chest pain lasting 40 min. ECG at that time revealed sub-millimeter ST elevations in V1 and V2, and T-wave inversions in V1-V3 (Fig. 1b). Given the patient’s recurring chest pain coronary angiography was performed. A spontaneous coronary artery dissection of the mid to distal left anterior descending artery was found with TIMI grade 3 flow into the distal part of the vessel (Fig. 2). The presentation of a long diffuse smooth section of intraluminal narrowing with 90% stenosis was consistent with Type 2a SCAD. No stent(s) were placed, and conservative management was elected which included continued daily aspirin and up-titration of Metoprolol. The next day the patient was transferred to the obstetrics service for further monitoring where she received two doses of intravenous betamethasone. Fetal growth ultrasound revealed normal and appropriate fetal growth, and the patient was observed for another 3 days. She remained asymptomatic from a cardiac stand-point and the rest of her hospital course was uneventful. The patient was discharged home in stable condition with strict precautions to return if chest pain were to recur.

## Outcome and follow up

The patient underwent a genetics consultation and after review of her history and pedigree it was determined that she had low concern for an underlying connective tissue disease. The patient returned to the state of her origin and re-established care with her original providers. Given the risk of recurrence of P-SCAD, she was counseled on various contraceptive options. With her history of prior myomectomy, she underwent a scheduled cesarean section delivery and bilateral tubal ligation without any complications. During the post-partum period, her original provider started her on calcitonin gene related peptide inhibitors as treatment for her migraines.

## Discussion and conclusions

The presentation of SCAD can range from chest pain to ventricular arrhythmias and even sudden cardiac death. P-SCAD is a diagnosis that should be strongly considered in pregnant patients presenting with chest pain given its risk for life-threatening complications for both the mother and fetus. P-SCAD has been shown to commonly involve the left anterior descending as well as the left main coronary artery, and in comparison, to SCAD in non-pregnant patients, it is more likely to involve multiple coronary arteries [3]. As a result, there is a higher incidence of a reduced ejection fraction, as well as more life-threatening complications. Yet there remains uncertainty regarding the underlying pathophysiology behind SCAD, and there is a lack of evidence regarding the ideal medical therapy for SCAD.

There are two hypotheses behind the pathophysiology of SCAD [4]. The first hypothesis is that an initial intimal tear within the coronary arterial wall serves to allow blood to enter the intimal space and therefore creates a false lumen susceptible to further dissection. In the second hypothesis, a spontaneous hemorrhage from the vasa vasorum within the arteriole wall causes an intramural hemorrhage, and the accumulation of this hematoma intramurally narrows the coronary artery lumen without the presence of a tear in the tunica intima. Interestingly it is proposed that the hormonal changes associated with pregnancy may lead to a weakened tunica media through impaired collagen synthesis and a change in mucopolysaccharide content [4]. This in turn puts the weakened coronary artery walls at greater risk of dissection, especially during periods of intense physical activity or stress, such as during labor. For our patient, hormonal therapy related to the IVF process in addition to psychological stress and anxiety related to infertility-treatment may have both further increased her risk for P-SCAD.

**Fig. 3:**
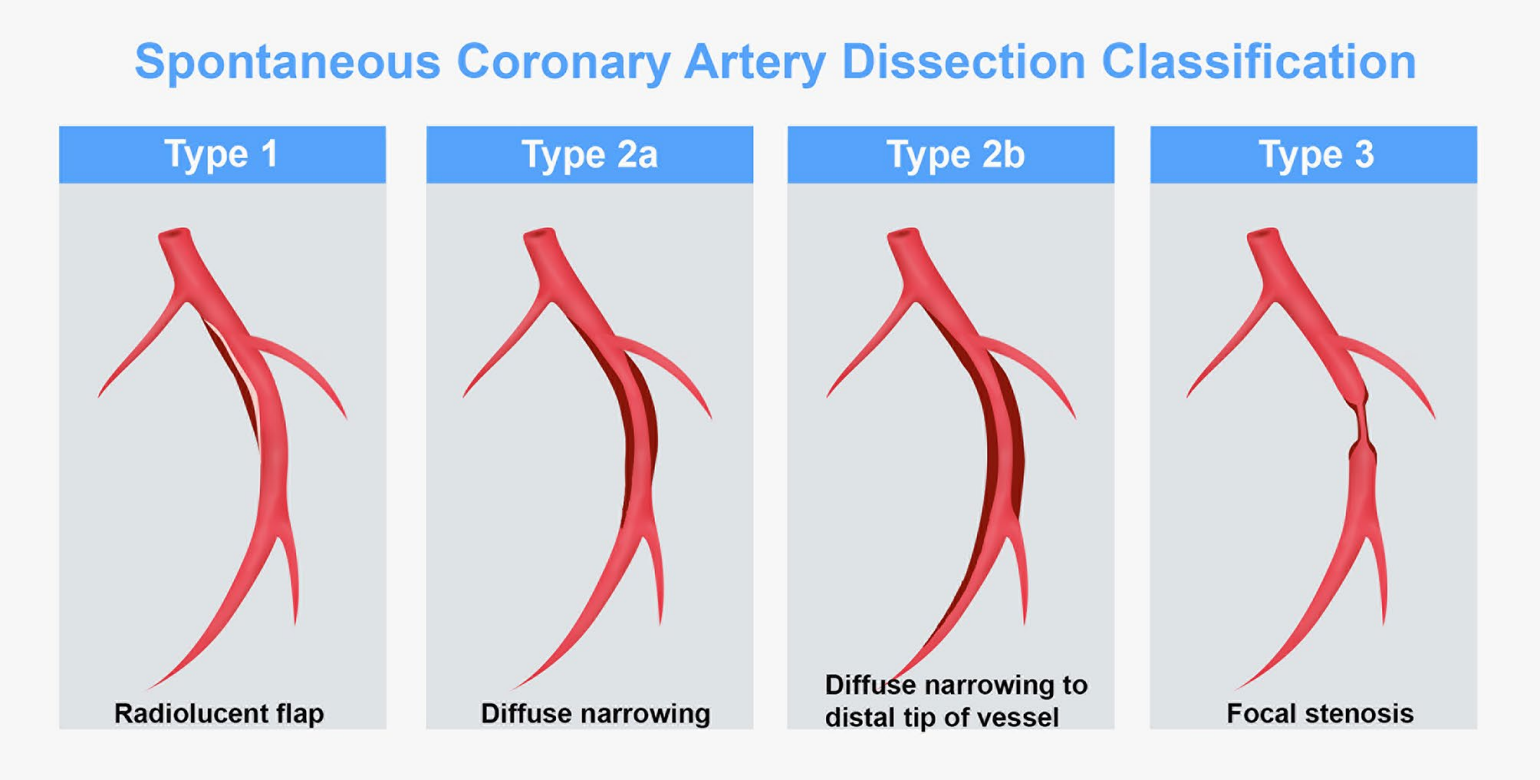
Spontaneous Coronary Artery Dissection Classification based on Angiographic Appearance

Based on the Yip-Saw classification, SCADs are categorized into three main types based on their various angiographic appearances (Fig. 3). Type 1 SCAD features multiple radiolucent lumens separated by a flap as a result of the contrast localizing into the false lumen(s). Type 2 SCAD features a long area of smooth stenosis, and are further delineated into types 2a and 2b. In type 2a the vessel regains caliber distal to the stenosed area. This categorization most closely resembles that seen in our patient’s angiographic appearance. In type 2b SCAD the stenosis continues to the most distally visible angiographical area. Type 3 SCAD lesions are characterized by a focal area of stenosis that can mimic atherosclerosis, and often requires intracoronary imaging with optical coherence tomography (OCT) or intravascular ultrasound (IVUS) for their identification.

Early diagnosis of SCAD is essential as it can guide treatment decisions. Additionally unnecessary pharmacological therapies can potentially cause more harm. Acute management of SCAD includes assessment with diagnostic coronary angiogram, especially since its management can deviate from that of standard ACS therapy. Thrombolytic agents should be avoided given the risk of adverse complications including extension of the dissection and hematoma. Percutaneous coronary intervention (PCI) in SCAD has been associated with an increased risk of complications such as iatrogenic dissection and abrupt vessel occlusion. PCI is generally avoided if there is minimal ischemia and coronary flow is preserved [3]. For the majority of SCAD patients, medical management is preferred, which consists of blood pressure control and standard heart failure medications when left ventricular dysfunction is present. For patients receiving stents, standard medical management is according to PCI guidelines. There is a lack of evidence to guide standardized medical therapy for SCAD; however, many patients have been managed with long-term aspirin given its reasonable side-effect profile. The addition of clopidogrel is of uncertain benefit in SCAD patients not undergoing PCI, and generally statin therapy is initiated only in patients with concomitant dyslipidemia. Given our patient’s overall hemodynamic stability, single vessel SCAD, and resolution of ischemia at time of angiography, conservative management with medications was seen as a reasonable approach. Given the majority of P-SCAD cases occur during the postpartum period, management of antepartum P-SCAD cases may vary as it will need to take into account fetal safety. For instance, the use of angiotensin-converting enzyme inhibitors in treating SCAD patients with left ventricular dysfunction may pose a significant risk of fetal malformation and complications in an antepartum patient. Additionally, it is important to note that the patient was started on calcitonin gene related peptide inhibitor therapy for her migraines. Her original provider was diligent in avoiding both triptans and ergot alkaloid medications out of concern that their vasoconstrictive and vasospastic properties could worsen her SCAD.

Currently there is a lack of randomized controlled trials to back a standardized medical management guideline for SCAD. Therefore, treatment should be individualized and based on a patient’s overall clinical picture and hemodynamic stability. For more stable patients with SCAD a conservative management approach should be preferentially considered. Given the high incidence of complications, both PCI and CABG should be performed for unstable patients, or in cases where benefits may outweigh the risks. When managing SCAD in pregnancy, it is important to consider when to deviate from common management practices for SCAD in non-pregnant patients. In comparison to most P-SCAD cases which occur post-partum, our case of medically managed P-SCAD contributes to the rare cases of antepartum SCAD in the literature, and reaffirms the importance of taking fetal safety into account. It is important to consider how hormonal exposure during IVF therapy may have contributed towards structural changes within the coronary artery walls, predisposing the patient to P-SCAD even months after IVF therapy. Patients interested in pursuing fertility treatment should be made aware of the potential cardiovascular risks associated with IVF therapy including P-SCAD, especially those who are receiving multiple rounds of IVF or have a family history of coronary artery disease. Future prospective studies regarding the cardiovascular outcomes of various management strategies of SCAD, as well as the association between IVF and SCAD cases may prove valuable in establishing further guidelines for the management of these patients.

## Data Availability

The datasets and imaging used and/or analyzed during the current study are available from the corresponding author on reasonable request.

## List of abbreviations

SCAD: Spontaneous Coronary Artery Dissection
P-SCAD: Pregnancy associated Spontaneous Coronary Artery Dissection
PCI: Percutaneous Coronary Intervention
CABG: Coronary Artery Bypass Grafting
IVF: In vitro fertilization
ECG: Electrocardiogram
